# An Experimental Investigation of Sexual Scripts by Partner Gender: Anticipated Clitoral Stimulation and Partner Orgasm Pursuit Shape Women’s Orgasm Expectations

**DOI:** 10.1007/s10508-025-03169-4

**Published:** 2025-06-17

**Authors:** Grace M. Wetzel, Carly Wolfer, Cheryl L. Carmichael, Diana T. Sanchez

**Affiliations:** 1 Department of Psychology, Rutgers, The State University of New Jersey, 53 Avenue E, Tillett Hall, Piscataway, NJ 08854-8040 USA; 2 Department of Psychology, The Graduate Center, The City University of New York, New York, NY USA; 3 Department of Psychology, Brooklyn College, The City University of New York, Brooklyn, NY USA

**Keywords:** Sexual scripts, Orgasm, Sexual orientation, Orgasm gap, Sexual orientation

## Abstract

Research has found that bisexual women expect clitoral stimulation and orgasm more when hypothetically partnered with another woman versus a man, which is consistent with the widely documented heterosexual orgasm gap. The current research aims to (1) investigate “perceived partner orgasm pursuit” (i.e., the participant’s perception that her partner is pursuing her orgasm) as a novel mechanism to explain this difference in orgasm expectations, and (2) demonstrate that manipulating the interpersonal sexual script with a male partner to include sufficient clitoral stimulation can increase orgasm expectations when women have sex with men. In Study 1, we found that bisexual or pansexual women (*N* = 457) partnered with a woman in a hypothetical scenario expected greater clitoral stimulation, partner pursuit of her orgasm, and likelihood of orgasm than those partnered with a man. In Study 2, bisexual or pansexual women (*N* = 362) who were partnered with a man in a hypothetical scenario that specified (1) clitoral stimulation or (2) opportunity to orgasm via her most reliable route expected her partner to pursue her orgasm and expected orgasm, at similar rates to women who were partnered with a woman whose sexual behavior was unspecified. On the contrary, women partnered with a man in a hypothetical scenario which specified vaginal intercourse only expected the lowest likelihood of orgasm. In both studies, condition predicted expected clitoral stimulation, which predicted perceived partner orgasm pursuit, which predicted orgasm expectations. This research suggests that sexual scripts explain differences in orgasm expectations by partner gender, and these expectations can be changed by shifting the sexual script at the interpersonal level to include sufficient opportunity for (and pursuit of) women’s orgasm via clitoral stimulation. Findings are discussed in the context of contributors to and solutions for the orgasm gap.

**Supplementary Information:**

The online version contains supplementary material available at 10.1007/s10508-025-03169-4.

## Introduction

Over the past decade, research continues to find that, on average, women experience orgasm less often than men during partnered sex (for reviews, see Mahar et al., 2020 and McElroy & Perry, 2024). For example, one key study found that heterosexual men reported experiencing orgasm 86% of the time with a familiar partner, compared to 62% of the time for heterosexual women (Garcia et al., 2014). While the magnitude of the orgasm gap differs across relationship contexts and is particularly exacerbated during casual sex (e.g., Armstrong et al., 2012; Wetzel & Sanchez, 2022), this gap persists throughout women’s lifetime (Gesselman et al., 2024). Orgasm frequency is associated with women’s sexual and relationship satisfaction, as well as general psychological well-being (Jones et al., 2018; Mahar et al., 2020; Rubin et al., 2019; Sprecher, 2002). Notably, women who have sex with women orgasm more often than women who have sex with men (e.g., Frederick et al., 2018), suggesting women’s attenuated orgasm frequency may be related to partner gender.

The purpose of the current project was to demonstrate that sexual scripts, or expectations for how a sexual encounter will unfold (Simon & Gagnon, 1986; Wiederman, 2015), contribute to partner gender differences in women’s orgasm. Using experimental vignettes, we demonstrate across two studies that (1) sufficient clitoral stimulation and (2) partner’s pursuit of women’s orgasm can help eliminate differences in orgasm expectations by partner gender, suggesting that sexual scripts at the interpersonal level are susceptible, effective targets for intervention to increase women’s orgasm frequency.

### Sexual Scripts and Partner Gender

Sexual scripts refer to sets of expectations for how a sexual encounter will unfold, based on a common social understanding and history of past experiences (Simon & Gagnon, 1986; Wiederman, 2015). The dominant sexual script for heterosexual partnered sex consists of foreplay, which is usually brief, followed by vaginal intercourse. The man is expected to orgasm from intercourse, after which sex is considered over (Mahar et al., 2020). This sexual script provides little opportunity for women’s orgasm via clitoral stimulation and instead prioritizes vaginal intercourse, which is associated with the lowest orgasm frequency for women, as compared to other combinations of sexual acts (e.g., receiving oral sex, intercourse paired with manual stimulation of the clitoris, etc.; Frederick et al., 2018; Mintz, 2017). While men’s orgasm frequencies do not differ by sexual orientation or partner gender (Frederick et al., 2018), women hold different sets of expectations for the encounter when their partner is a woman versus a man (Dickman et al., 2024).

Lesbian women may have a greater orgasm frequency than heterosexual women because their sexual encounters with other women follow a different (or less strict) sexual script (Lamont, 2017), without a phallocentric focus (Willis et al., 2018). For example, compared to women having sex with men, women having sex with women report a longer duration of their sexual encounters, which tend to include a greater variety of sexual acts that stimulate the clitoris (Blair & Pukall, 2014; Cohen & Byers, 2014; Schick et al., 2011, 2016). The sexual script for women having sex with other women likely includes more direct pursuit of women’s orgasm through a greater focus on clitoral stimulation (Cohen & Byers, 2014; Dickman et al., 2024; Frederick et al., 2018). Sex with a man and sex with another woman evoke different expectations for women (Dickman et al., 2024)—expectations which are importantly linked to orgasm pursuit and frequency (Wetzel et al., 2022, 2024). Therefore, differences in women’s orgasm across sexual orientation groups are likely driven, not by the partner’s gender alone, but by the very sexual scripts that govern the encounter. While sexual scripts are largely formed and function at the sociocultural level, they can be intervened on at the individual or interpersonal level. For example, couples have their own sexual script for how their sexual encounters typically unfold. To address the orgasm gap, researchers and theorists advise mixed-sex couples to adjust or adopt new sexual scripts in which sufficient clitoral stimulation is incorporated before, after, or during vaginal intercourse (Mintz, 2017; Wiederman, 2005, 2015); however, research has yet to empirically test these suggestions.

When studying heterosexual, gay, or lesbian populations, participant gender and partner gender are conflated and confounded, and sexual orientation differences likely mask differences that are, in fact, due to scripts associated with partner gender (Conley et al., 2014). Investigating bisexual women’s experiences provides a unique opportunity to explore the effect of partner gender on women’s sexual outcomes, because the gender of a hypothetical partner can be experimentally manipulated (Conley et al., 2014). In a small subsample, bisexual women reported a higher rate of experiencing orgasm in their real life when their casual sex partner was a woman than when their casual sex partner was a man (Eschler, 2004; Mahar et al., 2020). When with men, bisexual women may cater more to male partners' needs (i.e., less focus on their own orgasm) due to gendered orgasm entitlement (see Klein & Conley, 2021) and social norms that condition women to prioritize partner and relational goals over their own (Braun et al., 2003; McClelland, 2011; Wolfer & Carmichael, 2025). Research with a sample of bisexual women investigated the impact of partner gender and found that bisexual women did, in fact, have higher orgasm expectations when partnered with a woman compared to a man in a hypothetical scenario (Dickman et al., 2024).

#### Clitoral Stimulation

One explanation for women’s higher orgasm expectations when partnered with women compared to men is because of increased expectations for (and actual experiences of) clitoral stimulation, which is key to women’s orgasm (Dickman et al., 2024; Mahar et al., 2020; Mintz, 2017). Women are most likely to experience orgasm from sex acts that involve clitoral stimulation (e.g., oral sex, manual stimulation, vibrator use) and are more likely to orgasm from vaginal intercourse when intercourse includes simultaneous clitoral stimulation (Brewer & Hendrie, 2011; Frederick et al., 2018; Fugl-Meyer et al., 2006; Mintz, 2017; Salisbury & Fisher, 2014; Shirazi et al., 2018). While some women do report the ability to orgasm from vaginal intercourse alone, Mintz (2017) suggested that asking women about their most reliable route(s) to orgasm may be a more useful framework for understanding the sex acts that women need to experience orgasm. In a convenience sample, 96% of women listed clitoral stimulation alone or clitoral stimulation paired with vaginal penetration as their most reliable route to orgasm, while only 4% listed vaginal penetration alone (Mintz, 2017).

Women who have sex with women are more likely to engage in sexual activities beyond intercourse and define those activities as “sex” compared to women who have sex with men (Schick et al., 2011, 2016). Despite the established importance of clitoral stimulation for women’s orgasm, clitoral stimulation is often incidental in the heterosexual script (Wade et al., 2005). In work experimentally manipulating partner gender, bisexual women expected clitoral stimulation more when partnered with a woman than with a man in hypothetical scenarios, including greater expectations to receive oral sex, manual stimulation, and vibrator use (Dickman et al., 2024). Additionally, greater expectations for clitoral stimulation predicted greater orgasm expectations (Dickman et al., 2024). Thus, partner gender may alter the perceived likelihood of clitoral stimulation in the sexual script, which in turn, may shape orgasm expectations and frequency. Women should expect more clitoral stimulation when partnered with a woman than with a man, and manipulating the sexual script with a male partner to specify sufficient clitoral stimulation should increase orgasm expectations for women partnered with men.

#### Perceived Partner Orgasm Pursuit

Orgasm goal pursuit has been established as an important predictor of orgasm frequency for women, but not for men (Gusakova et al., 2020; Wetzel et al., 2024). In general, when one takes steps to strive for a desired outcome, that outcome becomes more likely (Rogers et al., 2015). The personal value placed on the outcome and expectations for its achievement guide goal pursuit (i.e., expectancy-value theory; Wigfield & Eccles, 2000). Thus, women pursue orgasm more strongly when they value and expect orgasm (Wetzel et al., 2024), and women’s orgasm value and expectations are shaped by their existing orgasm frequency or orgasm history (Blumenstock, 2022; Wetzel et al., 2022). Research with heterosexual, lesbian, and bisexual women found that clitoral stimulation and orgasm expectations predicted women’s orgasm goal pursuit. Women who experienced or expected clitoral stimulation were more likely to expect orgasm, and, in turn, reported greater pursuit of orgasm, in both real life and hypothetical encounters (Dickman et al., 2024).

Importantly, however, orgasm pursuit is an interdependent rather than an individual process, meaning relational dynamics play an important role in orgasm goal pursuit (Wolfer & Carmichael, 2025). We define “perceived partner orgasm pursuit,” in this case, as the participant’s perception that her partner is pursuing her orgasm. Data from a 21-day diary of heterosexually partnered individuals (Wolfer & Carmichael, 2025) demonstrates a gap in orgasm pursuit that may be partially responsible for the orgasm gap: men and women disproportionately pursued the male partner’s orgasm. One’s own orgasm pursuit and perceived partner orgasm pursuit were associated with orgasm likelihood and sexual satisfaction. Even more, the effect of personal orgasm pursuit on orgasm and sexual satisfaction was positive and significant only at high levels of perceived partner orgasm pursuit, suggesting that a woman’s personal efforts to orgasm with a male partner are effective *only* when met with perceived collaboration from a partner. These results underline the importance of examining orgasm pursuit and the orgasm gap from an interpersonal lens, rather than utilizing only individual-level determinants and outcomes.

Because clitoral stimulation is vital for most women to orgasm, the extent to which a woman expects clitoral stimulation to be included in the sexual script likely affects the extent to which she expects her partner to actively pursue her orgasm. Clitoral stimulation often requires active effort from one’s partner, such as when receiving oral sex or manual stimulation. So, when a woman expects that clitoral stimulation will be part of the sexual script, she likely expects that her partner will put more effort toward the pursuit of her orgasm, by engaging in those clitoral sex acts needed for her orgasm.

### Current Project

The purpose of the current project was to demonstrate that sexual scripts with differing levels of clitoral stimulation and perceived partner orgasm pursuit drive partner gender differences in expected orgasm likelihood, rather than stable differences in men’s (versus women’s) ability to please a female partner. We predict that sexual scripts can be manipulated at the interpersonal level to eliminate women’s attenuated orgasm expectations with a male partner, and we point to two actionable steps that are lacking, but can importantly be amplified, in the heterosexual script to remedy the orgasm gap: clitoral stimulation and partner pursuit of women’s orgasm.

To investigate the effect of partner gender on orgasm, we examined the expectations bisexual women hold for a sexual encounter when experimentally partnered with a woman versus a man. According to existing research, bisexual women’s script for sex with another woman should include greater engagement with clitoral sexual acts to help her achieve orgasm, while her script for sex with a man should be characterized by a gendered orgasm pursuit gap (Wolfer & Carmichael, 2025), brief clitoral stimulation during foreplay, and a focus on vaginal intercourse (Mahar et al., 2020; Mintz, 2017). Thus, women should experimentally differ in their orgasm expectations by partner gender, as a result of these accompanying sexual scripts—specifically, clitoral stimulation and perceived partner orgasm pursuit.

These behavioral and motivational variables, which should help to explain the partner gender differences in orgasm expectations, are notably modifiable, rather than fixed, elements of partnered sexual scenarios. Thus, in Study 2, we manipulated the interpersonal sexual script with male partners in order to investigate whether specifying sufficient clitoral stimulation and/or opportunity for women’s orgasm with a male partner could eliminate this partner gender difference in orgasm expectations.

### Study 1

#### Hypotheses

**Fig. 1:**
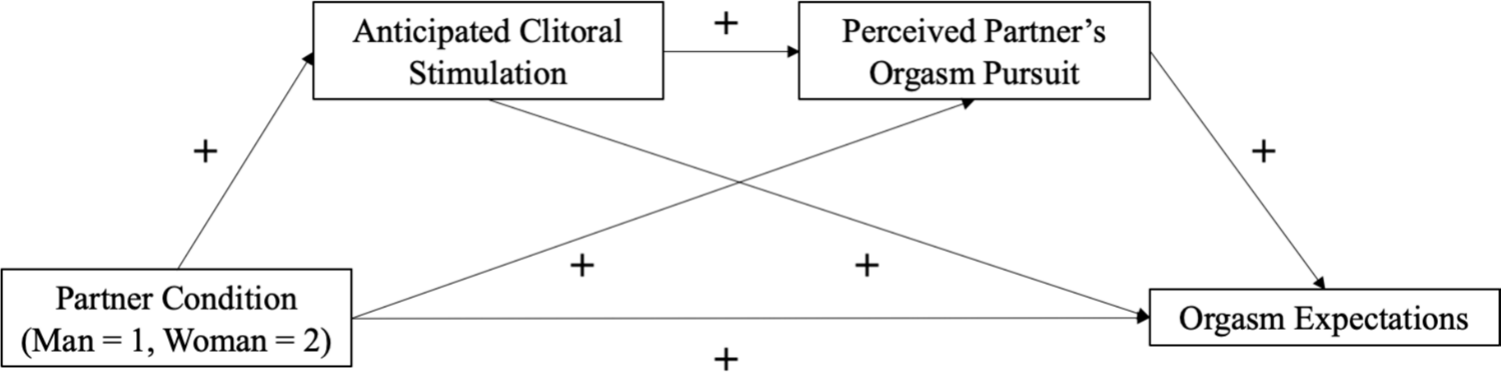
Proposed model in which the relationship between partner gender and orgasm expectations is mediated by anticipated clitoral stimulation and perceived partner orgasm pursuit

In Study 1, we predicted that bisexual women would hold greater expectations for clitoral stimulation, perceived partner orgasm pursuit, and orgasm itself when hypothetically partnered with a woman compared to a man. We predicted that anticipated clitoral stimulation and perceived partner orgasm pursuit would mediate the relationship between partner gender and orgasm expectations (Fig. 1).

We tested a serial mediation in which we predicted that greater expectations for clitoral stimulation would predict greater perceived partner orgasm pursuit, which would help to explain the greater orgasm expectations for women when partnered with women versus men. We conceptualize orgasm goal pursuit as a motivational variable (i.e., goal setting and striving) and clitoral stimulation as a behavioral variable (i.e., engagement in actual sex acts). While motivation generally informs behavior (i.e., a person’s own intention to pursue orgasm would predict engagement in specific sex acts), women’s *perception* of her partner’s motivation is likely *preceded* by her partner’s behavior during a sexual encounter. That is, if a partner engages in clitoral stimulation, a woman is likely to perceive that her partner is actively pursuing her orgasm, which in turn should be associated with increased orgasm expectations (and by extension, orgasm frequency). Thus, we hypothesized that expected clitoral stimulation would predict perceived partner pursuit in a serial mediation. However, we also tested the mediators in reverse order for comparison.

## Method

### Participants and Procedure

We recruited an online sample of cisgender, bisexual or pansexual women who were at least 18 years old and had been sexually active in the past year. Using G*Power analysis, to detect a small effect size of 0.30 with 85% power, for comparisons between two independent groups, a sample of 402 participants would be needed. Thus, we aimed to recruit up to 500 participants to account for participant loss and exclusion; 467 eligible participants completed the full study. Ten participants were excluded for incorrectly answering the manipulation check, and another participant was excluded for incorrectly answering more than one attention check. Our final sample consisted of 457 participants, which we recruited across two online platforms that are commonly used for research participation, Prolific (62%; *n* = 284) and ResearchMatch (38%; *n* = 173). On the Prolific recruitment platform, participants were compensated $1.39 for completing a median-length 8-min study ($10.43/hour on average). In order to reach the desired sample size, we recruited additional participants on the ResearchMatch platform, on which participants completed the study on a volunteer basis (i.e., without compensation). Participant demographics can be found in Table 1.

**Table 1:** Demographic information for Studies 1 and 2

	Study 1	Study 2
*N*	457	362
Age *M* (*SD*)	32.48 (10.20)	33.43 (10.11)
Race/Ethnicity *n* (%)		
White	373 (81.6)	292 (80.7)
Latinx	45 (9.8)	36 (9.9)
Black/African American	38 (8.3)	38 (10.5)
Asian	28 (6.1)	27 (7.5)
Native American/Alaska Native	9 (2.0)	11 (3.0)
Middle Eastern	5 (1.1)	5 (1.4)
Hawaiian/Pacific Islander	3 (0.7)	2 (0.6)
Multiracial	16 (3.5)	17 (4.7)
Another identity not listed	1 (0.2)	1 (0.3)
Prefer not to disclose	3 (0.7)	0 (0.0)
Relationship Status *n* (%)		
Yes	376 (82.3)	271 (74.9)
No	69 (15.1)	79 (21.8)
Other	12 (2.6)	12 (3.3)
Medication *n* (%) Yes	93 (20.4)	96 (26.5)

Only cisgender, bisexual or pansexual women were recruited for both studies. Relationship status asked if participants were currently in a relationship in their real life. Medication refers to participants who reported taking any medications or having a medical condition that interferes with their ability to experience orgasm

After completing eligibility screening, participants were randomly assigned to a partner gender condition, were asked to read a hypothetical sexual scenario, and then completed measures in the order presented below. Participants completed demographic questions last, received debriefing, and exited the study. Additional measures included for exploratory purposes are not reported in the current manuscript (view full surveys, data, and syntax for both studies at https://osf.io/qhd5n/).

### Measures

#### Hypothetical Sexual Scenario

Participants were given the following instructions: “Regardless of your current relationship status, please imagine yourself in the context of the following hypothetical scenario.” Participants were randomly assigned to one of two conditions which varied the gender of the hypothetical partner in the vignette:

You are out to dinner with a [woman/man] who you feel comfortable with and find very attractive. You’ve been seeing this [woman/man] for a while and have an established sexual relationship with [her/him]. When you get home from dinner, things start heating up and you make your way to the bedroom together. You are in the mood and are looking forward to beginning sexual activity with [her/him].

Participants were asked to keep in mind the described sexual encounter when responding to the below measures. After completing all measures, participants were asked to recall the gender of their hypothetical partner in the scenario as a manipulation check. The wording of this vignette was adapted from previous research (Dickman et al., 2024; Wetzel et al., 2024).

#### Expected Sex Acts

Participants were asked how much they expected to experience each of 18 sex acts in the encounter described (adapted from Frederick et al., 2018 and Dickman et al., 2024; e.g., receive oral sex from partner) on a scale from 1 (*definitely would not experience*) to 7 (*would definitely experience*). These items were presented in random order; the full list of sex acts can be found in Table 2.

**Table 2:** Comparisons by hypothetical partner gender (Study 1)

Study measure	Range	Man		Woman					
		*Mean*	*SD*	*Mean*	*SD*	*t*	*df*	*p*	*d*
Orgasm value	1–7	5.05	1.35	4.87	1.30	1.46	455	.146	0.14
Orgasm expectations	1–7	4.81	1.40	5.69	1.14	− 7.36	440.31	** < .001**	− 0.69
Orgasm goal pursuit	1–7	5.44	1.00	5.62	0.99	− 1.91	455	.057	− 0.18
Pursuit of Partner’s Orgasm	1–7	6.47	0.54	6.34	0.60	2.27	455	**.024**	0.21
Perceived Partner Orgasm Pursuit	1–7	5.53	0.98	6.01	0.73	− 5.97	425.82	** < .001**	− 0.56
Anticipated Clitoral Stimulation	1–7	5.06	0.97	5.73	0.82	− 7.92	455	** < .001**	− 0.74
Sex Acts									
Vaginal intercourse with simultaneous clitoral stimulation*^+^	1–7	5.34	1.39	5.17	1.56	1.20	446.44	.232	0.11
Vaginal intercourse without simultaneous clitoral stimulation^+^	1–7	5.50	1.58	4.18	1.78	8.35	446.48	** < .001**	0.78
Anal Intercourse^+^	1–7	2.08	1.32	2.23	1.42	− 1.18	455	.237	− 0.11
Give oral sex	1–7	5.54	1.41	6.05	1.07	− 4.39	428.60	** < .001**	− 0.41
Receive oral sex*^+^	1–7	5.08	1.42	5.97	1.08	− 7.55	428.61	** < .001**	− 0.70
Manual stimulation of partner’s genitals	1–7	6.01	1.09	6.44	0.75	− 4.95	455	** < .001**	− 0.46
Receive manual stimulation of clitoris from partner*^+^	1–7	5.82	1.01	6.35	0.78	− 6.27	430.74	** < .001**	− 0.59
Receive manual stimulation of vagina from partner^+^	1–7	5.73	1.11	6.29	0.91	− 5.89	441.27	** < .001**	− 0.55
Gentle Kissing	1–7	5.90	1.26	6.38	0.81	− 4.85	392.39	** < .001**	− 0.45
Deep Kissing	1–7	6.23	1.13	6.51	0.78	− 3.07	409.95	**.002**	− 0.29
Use of a vibrator or other sex toy on clitoris*^+^	1–7	4.02	1.68	5.43	1.38	− 9.83	440.99	** < .001**	− 0.92
Use of a vibrator or other sex toy in vagina^+^	1–7	3.48	1.62	5.17	1.40	− 11.95	448.45	** < .001**	− 1.12
Use of a vibrator or other sex toy on partner	1–7	2.94	1.57	5.35	1.34	− 17.66	446.62	** < .001**	− 1.65
Sexual touching or oral stimulation to body parts other than genitals (e.g., nipples, neck, thighs, etc.)^+^	1–7	6.21	1.02	6.50	0.80	− 3.46	434.58	** < .001**	− 0.32
Sexual touching or oral stimulation to partner’s body parts other than genitals (e.g., nipples, neck, thighs, etc.)	1–7	6.06	1.06	6.52	0.82	− 5.12	431.78	** < .001**	− 0.48
Stimulate partner’s anus with hands or mouth	1–7	2.19	1.44	2.85	1.77	− 4.39	432.31	** < .001**	− 0.41
Receive anal stimulation from partner’s hands or mouth^+^	1–7	2.78	1.71	3.13	1.98	− 2.01	443.01	**.045**	− 0.19
Partner would masturbate in front of me with their hands	1–7	3.78	1.59	4.75	1.41	− 6.91	455	** < .001**	− 0.65
Masturbate in front of partner with hands^+^	1–7	3.97	1.67	4.63	1.63	− 4.30	455	** < .001**	− 0.40

^*^Indicates the four items included in the clitoral stimulation composite. ^+^Indicates the eleven items that were included in the “Reliable Route to Orgasm” measure. Bolded *p*-values indicate *p* < .05

#### Anticipated Clitoral Stimulation

We created an anticipated clitoral stimulation variable (Dickman et al., 2024) by averaging participants’ scores for four of the sex acts: vaginal intercourse with simultaneous clitoral stimulation, receive oral sex from partner, receive manual stimulation of the clitoris from partner, and use of a vibrator or other sex toy on the clitoris.

#### Orgasm Goal Pursuit

We administered three separate orgasm goal pursuit measures (adapted from Gusakova et al., 2020; Wolfer & Carmichael, 2025) which captured participants’ intent to (1) pursue their own orgasm, (2) pursue their partner’s orgasm, and (3) their perception that their partner would pursue their orgasm, in the scenario described. Each measure adapted the same six items presented on a scale from 1 (*strongly disagree*) to 7 (*strongly agree*). Sample items for personal orgasm goal pursuit include, “My goal would be to orgasm” and “I would try to have an orgasm” (α = 0.83). For partner pursuit, these two items instead were, “My goal would be to help my partner orgasm” and “I would try to help my partner have an orgasm” (α = 0.82). For perceived partner orgasm pursuit, these items read, “My partner’s goal would be to help me orgasm” and “My partner would try to help me have an orgasm” (*α* = 0.89).

#### Orgasm Value and Expectations

We captured participants’ orgasm value in the sexual encounter described by combining a standard orgasm importance item (adapted from Gusakova et al., 2020; “Orgasm would be important to my sexual satisfaction”) with a three-item value subscale (adapted from Major & Schmader, 1998; e.g., “I would care a great deal about experiencing orgasm with this partner”). These four items were assessed on a scale from 1 (*strongly disagree*) to 7 (*strongly agree*) and averaged such that higher scores indicated greater orgasm value (*α* = 0.89). Participants’ orgasm expectations were measured using the following item (adapted from Blumenstock, 2022) on a scale from 1 (*definitely would not experience*) to 7 (*would definitely experience*): “In the sexual encounter described, how much do you expect to experience orgasm?”.

#### Reliable Route to Orgasm

Finally, we asked participants to identify their most reliable route(s) to orgasm in their real life, which was defined for participants as the sexual act(s) that result in orgasm most efficiently and reliably for them when with a sexual partner. Participants could select more than one item from a checklist of 11 sexual acts (Table 3), as well as an open-text “other” option. We included this question to ensure that our empirical focus on four specific clitoral sex acts reflected the reality of our female participants’ most reliable orgasm experiences.

**Table 3:** Women’s reports of their most reliable route(s) to orgasm (Studies 1 and 2)

Sex act	Participants who selected yes *n* (%)	
	Study 1	Study 2
Use of a vibrator or other sex toy on your clitoris*	307 (67.2)	263 (72.7)
Vaginal intercourse with simultaneous clitoral stimulation*	269 (58.9)	235 (64.9)
Receive manual stimulation of your clitoris from partner (i.e., my partner’s hand(s) would stimulate my clitoris)*	211 (46.2)	173 (47.8)
Receive oral sex from partner*	210 (46.0)	175 (48.3)
Sexual touching or oral stimulation to my body parts other than genitals (e.g., nipples, neck, thighs, etc.)	135 (29.5)	84 (23.2)
Receive manual stimulation of your vagina from partner (i.e., my partner’s hand(s) would stimulate my vagina)	130 (28.4)	109 (30.1)
Use of a vibrator or other sex toy in your vagina	108 (23.6)	88 (24.3)
I would masturbate in front of my partner with my hands	101 (22.1)	88 (24.3)
Vaginal intercourse without simultaneous clitoral stimulation	79 (17.3)	53 (14.6)
Receive anal stimulation from my partner’s hands or mouth	29 (6.3)	22 (6.1)
Anal intercourse	21 (4.6)	24 (6.6)
Other	13 (2.8)	15 (4.1)

Participants could select more than one sex act. Sex acts are listed in descending order by frequency for Study 1. * indicates sex acts that were included in the clitoral stimulation composite measure

### Analytic Strategy

After assessing reliability, creating composite variables, and running initial correlations (see Table 4), we conducted a series of independent samples *t*-tests to compare participants by condition (partner gender: man vs. woman) on all study variables (Table 2). Using PROCESS software (Hayes, 2023), we conducted a serial mediation model. We included women’s own orgasm goal pursuit as a covariate in the serial mediation, to demonstrate that the effect of perceived partner orgasm pursuit was distinct from one’s own orgasm pursuit.

**Table 4:** Correlations for main study variables (Studies 1 and 2)

	Condition	Orgasm goal pursuit	Perceived partner orgasm pursuit	Pursuit of partner’s orgasm	Expected clitoral stimulation	Orgasm expectations	Orgasm value	Age
Condition	–	.20***	.31***	.03	.22***	.28***	.11*	− .04
Orgasm Goal Pursuit	.09	–	.50***	.31***	.38***	.61***	.64***	.06
Perceived Partner Orgasm Pursuit	.27**	.48***	–	.35***	.59***	.61***	.28***	.00
Pursuit of Partner’s Orgasm	− .11*	.40***	.43***	–	.18***	.15**	.19***	− .07
Anticipated Clitoral Stimulation	.35***	.37***	.47***	.21***	–	.40***	.19***	− .07
Orgasm Expectations	.33***	.66***	.50***	.15**	.38***	–	.35***	.03
Orgasm Value	− .07	.67***	.23***	.25***	.16***	.48***	–	.01
Age	.04	.17***	.08	− .01	.03	.20***	.22***	–

Study 1 Correlations are presented below the diagonal; Study 2 correlations are presented above the diagonal. For Study 1 Condition: 1 = Male partner, 2 = Female partner. For Study 2 Condition: 1 = Man—Intercourse Only, 2 = Woman—Comparison, 3 = Man—Clitoral Stimulation, 4 = Man—Reliable Route. *** *p* < .001; ** *p* < .01; * *p* < .05

## Results

### Reliable Route to Orgasm

After conducting frequency analyses on all 12 sex acts included in the reliable route measure (Table 3), we found that women’s four most reliable routes to orgasm with a partner were the same four items included in our clitoral stimulation composite: use of a vibrator/sex toy on clitoris (67.2% of participants), vaginal intercourse with simultaneous clitoral stimulation (58.9%), receive manual stimulation of clitoris (46.2%) and receive oral sex (46.0%). There was a significant drop in frequency for the next most popular response (stimulation of other body parts; 29.5%), *χ*^2^ (1, 457) = 22.32, *p* < 0.001. These results provide support for the use of the selected items in the clitoral stimulation composite measure, as well as justification that this clitoral stimulation measure assesses the sex acts needed in the sexual script to facilitate most women’s orgasm.

### Differences by Partner Gender

**Fig. 2:**
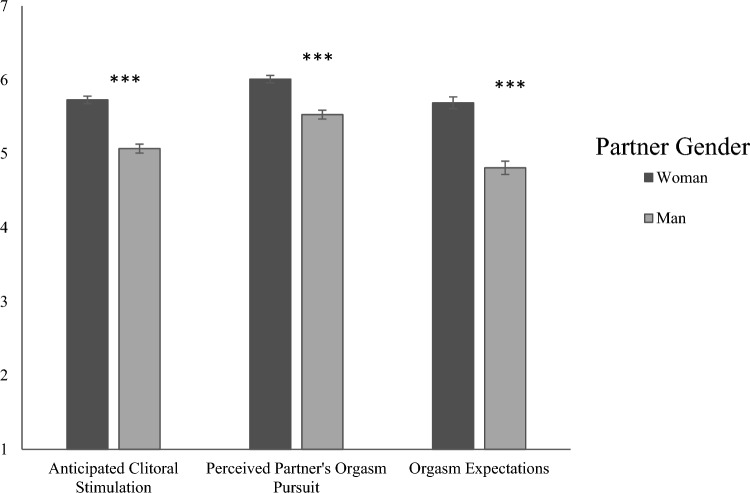
Comparisons by partner gender for main dependent variables (Study 1). *Note* * *p* < .05; ** *p* < .01; *** *p* < .001. Error bars ± 1 SE

When partnered with a woman, bisexual women reported greater expectations for clitoral stimulation, greater perceived partner orgasm pursuit, and greater orgasm expectations than when partnered with a man, as predicted, *ps* < 0.001 (Fig. 2; Table 2). All of these partner gender differences had moderate-to-large effect sizes (Cohen’s *d*s > 0.55), as Cohen’s *d* values above 0.5 are generally considered to indicate a moderately-sized effect (Cohen, 1988).

Additionally, women reported greater pursuit of their partner’s orgasm when partnered with a man than when partnered with a woman, but did not differ in their own orgasm goal pursuit or their orgasm value based on partner gender. In terms of specific sex acts, women expected vaginal intercourse *without* simultaneous clitoral stimulation to be more likely when partnered with a man, but expected most other sex acts to be more likely when partnered with a woman. Degrees of freedom were adjusted when analyses did not pass Levene’s test for equality of variances. See Table 2 for the full list of *t*-test comparisons.

### Serial Mediation Model

**Fig. 3:**
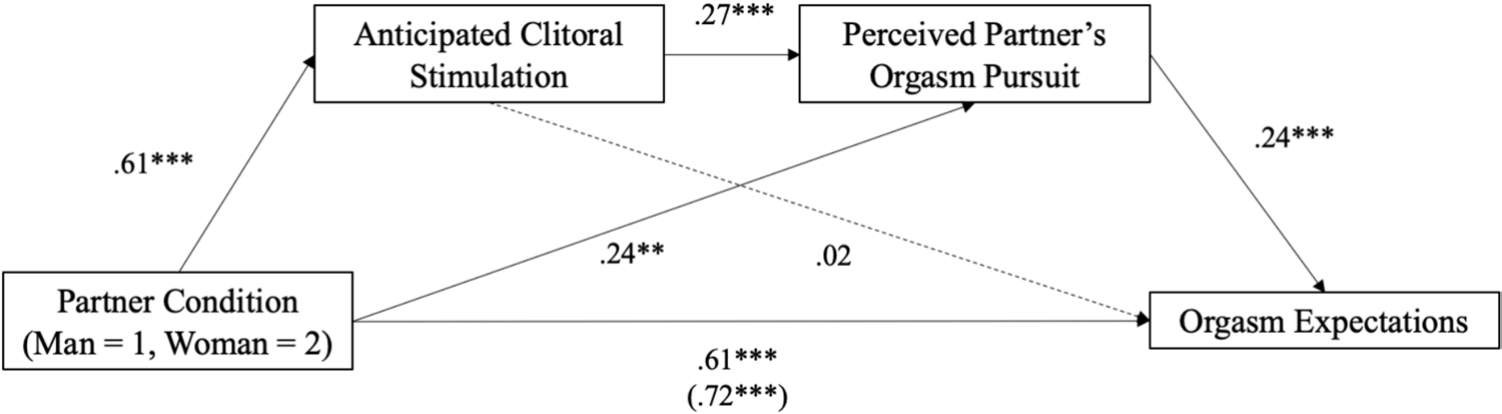
Serial mediation predicting orgasm expectations (Study 1). *Note* Serial indirect effect = .04 [.01, .07]. Analysis controlled for women’s own orgasm pursuit. Unstandardized values are shown

Using 5,000 bootstrapped samples, the serial mediation was significant; serial indirect effect = 0.04 [95% CI: 0.01, 0.07], *SE* = 0.01. Partner gender condition predicted expected clitoral stimulation, such that being partnered with a woman (versus a man) predicted greater expectations for clitoral stimulation, *p* < 0.001. Greater expectations for clitoral stimulation predicted greater perceived partner orgasm pursuit, *p* < 0.001, which predicted greater orgasm expectations, *p* < 0.001. See Fig. 3 for coefficients. One’s own orgasm pursuit was a significant covariate, *ps* < 0.001. For the alternative serial mediation with perceived partner orgasm pursuit as the initial mediator predicting anticipated clitoral stimulation, the model was not significant (see Supplement Document).

### Discussion (Study 1)

In Study 1, we found that bisexual women’s sexual expectations differed based on the gender of a hypothetical partner. When partnered with other women, bisexual women had greater expectations for clitoral stimulation, perceived partner orgasm pursuit, and orgasm likelihood, than when partnered with men. Women’s greater expectations for orgasm when partnered with women versus men were explained, at least in part, by the associated change in anticipated clitoral stimulation and perceived partner orgasm pursuit.

These findings align with sexual script theory, which suggests that sexual expectations and behaviors are shaped by scripts at multiple levels: sociocultural, interpersonal, and individual (Simon & Gagnon, 1984, 1986; Wiederman, 2005). Sociocultural scripts prescribe default expectations for how a sexual encounter should or would normally go: for women partnered with men, that includes vaginal intercourse and pursuit of men’s orgasm (Klein & Conley, 2021; Mahar et al., 2020), and for women partnered with women, that includes more clitoral stimulation and shared pleasure pursuit (Frederick et al., 2018; Schick et al., 2016). Although deeply rooted and challenging to change at the cultural and institutional level, sociocultural scripts are not entirely prescriptive or predictive of individual behavior across specific interpersonal scenarios (Simon & Gagnon, 1984, 1986; Wiederman, 2005). Instead, individuals and their partners usually adapt sociocultural scripts based on the particulars of each encounter or relationship, creating individual and interpersonal scripts that require iterative “modification and improvisation of previously adopted scripts” based on new situational elements (Wiederman, 2005, p. 8).

Thus, while women partnered with men may, by default, expect sex to follow a dominant sociocultural script, it may be possible to increase orgasm expectations by modifying specific situational elements, such as clitoral stimulation and perceived partner orgasm pursuit. If adding these elements into the sexual situation with men eliminated partner gender differences in orgasm expectations, these results would provide a concrete mechanism for men partnered with women to adopt to improve women’s orgasm expectations and outcomes in real life.

### Study 2

The purpose of Study 2 was to confirm that partner gender differences in orgasm expectations are, in fact, informed by the prototypical sociocultural script when a woman engages in sex with a man versus a woman, by manipulating that script at the interpersonal level (i.e., with a particular partner). We focus on manipulating clitoral stimulation and partner pursuit of women’s orgasm because these are markedly lacking in the prototypical heterosexual script, yet are critical for orgasm equity. While the prototypical heterosexual script deems vaginal intercourse the main and most important sex act that signposts the beginning and end of a sexual encounter (Mahar et al., 2020; McPhillips et al., 2001; Randall & Byers, 2003; Willis et al., 2018), intercourse alone is associated with the lowest orgasm frequency for women (Frederick et al., 2018). Direct clitoral stimulation, on the other hand, has long been shown to be essential for most women to reliably experience orgasm, despite its de-prioritization in the dominant heterosexual script (Mahar et al., 2020). Sufficient pursuit of women’s orgasm by the male partner is also linked to women’s orgasm frequency, but women’s perception of their male partner’s pursuit is noticeably low compared to women’s pursuit of men’s orgasm (i.e., orgasm pursuit gap; Wolfer & Carmichael, 2025). This set of findings suggests that intervening on this gendered orgasm *pursuit* gap and the de-prioritization of clitoral stimulation may be promising, but untapped, pathways for increasing women’s orgasm expectations (and frequency) when with a male partner. We seek to investigate whether editing the heterosexual script with a particular hypothetical partner to include sufficient clitoral stimulation and/or pursuit of women’s orgasm will increase women’s orgasm expectations.

We manipulated the sexual script in the scenario with a male sexual partner to differ based on the presence of intercourse and clitoral stimulation. Specifically, we presented a script which specified intercourse only, a script which specified consistent inclusion of clitoral stimulation, and a script which specified inclusion of the sex acts that most reliably result in orgasm for the participant. This third condition was included to signal opportunity for the woman’s orgasm via her most reliable route, as opposed to general clitoral stimulation, because the most reliable route to orgasm differs between women (Mintz, 2017; as seen in Study 1). Finally, we presented the same female partner condition used in Study 1 as a comparison condition in Study 2. This comparison condition allowed us to test whether bisexual women’s orgasm expectations with a male partner can become commensurate with their expectations with a female partner (whose behavior is unspecified).

#### Hypotheses

In Study 2, we predicted that women in the intercourse only condition with a male partner would report lower perceived partner orgasm pursuit and orgasm expectations than women in the other three conditions. In other words, we hypothesized that if we altered the sexual script with a male partner to specify sufficient clitoral stimulation, we could match women’s orgasm expectations to those with a female partner whose behavior was unspecified. We expected that the relationship between condition and orgasm expectations would function through the same mediator as Study 1: perceived partner orgasm pursuit.

Importantly, we attempted to manipulate clitoral stimulation as a mechanism in Study 2, so we did not include this variable in the mediation model in Study 2. In the conditions wherein clitoral stimulation was specified in the script (via clitoral stimulation broadly or via the woman’s most reliable route), the assessment of anticipated clitoral stimulation served as a manipulation check.

## Method

### Participants and Procedure

For Study 2, we recruited an online sample of cisgender, bisexual or pansexual women who were at least 18 years old and currently residing in the United States from the same two online recruitment platforms as Study 1: ResearchMatch (65.2%) and Prolific (34.8%). Using G*Power analysis, to detect a small effect size of 0.25 with 85% power, for comparisons between four independent groups in a one-way ANOVA, a sample of 299 participants would be needed. The full study was completed by 397 eligible participants, and we excluded 35 participants for incorrectly answering a manipulation check question. No participants answered more than one attention check incorrectly. Thus, our final sample size consisted of 362 participants. On ResearchMatch, participants completed the study on a volunteer basis. An additional sample of participants was recruited on Prolific in order to reach the desired sample size. On the Prolific recruitment platform, participants were compensated $1.30 for their completion of a median-length 8-min study ($9.32/hour on average). Some variables differed by data source (see Supplement Document). Participant demographics can be found in Table 1.

After completing screening, participants were randomly assigned to one of four conditions, were asked to read a hypothetical sexual scenario, and completed the measures below in the order presented. Finally, participants completed demographic questions and received debriefing.

### Measures

#### Hypothetical Sexual Scenario

We attempted to manipulate interpersonal sexual scripts in an initial iteration of Study 2, but anticipated clitoral stimulation did not differ between the three male partner conditions. Thus, our initial vignettes did not successfully manipulate clitoral stimulation within the sexual script. We revised the vignettes based on these results to create the vignettes used in the current study. Vignettes and results for this pilot version of Study 2 can be found in the Supplement Document.

Participants were given the same instructions as in Study 1, and were randomly assigned to read one of four vignettes. The “Woman—Unspecified” condition was exactly the same as the female partner condition used in Study 1. The “Man—Intercourse Only” condition included an additional sentence specifying details about the sexual script with the hypothetical partner, presented here in bold text:

You are out to dinner with a man who you feel comfortable with and find very attractive. You’ve been seeing this man for a while and have an established sexual relationship with him. When you get home from dinner, things start heating up and you make your way to the bedroom together. **In the past, you and this partner have typically started and ended your sexual encounters with vaginal intercourse only.** You are in the mood and are looking forward to beginning sexual activity with him.

The “Man—Clitoral Stimulation” condition included the following sentence instead: “In the past, you and this partner have typically engaged in a range of sexual activities in your sexual encounters, always including clitoral stimulation.” The “Man—Reliable Route” condition included the following sentence instead: “In the past, you and this partner have typically engaged in a range of sexual activities in your encounters to ensure you both have the opportunity to orgasm in the way that you experience orgasm most efficiently and reliably.”

Participants were asked to complete the below measures in response to the sexual encounter described. At the end of the study, we asked two manipulation check questions to ensure that participants could correctly identify the vignette that they were assigned. First, we asked participants to identify the gender of their hypothetical partner. Then, for participants in the three male partner conditions, we asked them to identify the sentence which came from their scenario, from a list of three sentence options which were unique to each of the three conditions. Only participants who responded to these questions correctly were included in the final sample.

#### Orgasm Goal Pursuit and Orgasm Expectations

Participants responded to the same three orgasm goal pursuit measures in Study 2 as in Study 1: personal orgasm goal pursuit (*α* = 0.85), pursuit of their partner’s orgasm (*α* = 0.80), and perceived partner orgasm pursuit (*α* = 0.90). Participants completed the same orgasm expectations measure as in Study 1. These two measures were counterbalanced.

#### Orgasm Value

Participants responded to the same four-item orgasm value measure as in Study 1 (*α* = 0.87).

#### Expected Sex Acts

Participants were asked how much they expected to experience each of a selection of sex acts in the sexual encounter described in the same manner as Study 1.

##### Anticipated Clitoral Stimulation

We created an anticipated clitoral stimulation measure using a composite score of the same four sex acts as in Study 1.

#### Most Reliable Route to Orgasm

At the end of the survey, we again asked participants to identify their most reliable route(s) to orgasm in their real life, which was defined in the same way, with the same response options, as Study 1 (Table 3).

### Analytic Strategy

After running reliability and correlation analyses (Table 4) and creating composites, we conducted a series of one-way ANOVAs to compare participants by condition (Woman—Unspecified, Man—Intercourse Only, Man—Clitoral Stimulation, and Man—Reliable Route) on all relevant study variables (Table 5). Then, we conducted the proposed mediation model in which condition predicted orgasm expectations via the associated change in perceived partner orgasm pursuit. We tested this multi-categorical mediation model using PROCESS software (Hayes, 2023), and used the Man—Intercourse Only condition, from which the other three conditions differed, as the reference group. Thus, the Man—Intercourse Only condition was directly compared to each of the other conditions in turn. We again controlled for women’s own orgasm goal pursuit by including it as a covariate.

**Table 5:** Comparisons by condition (Study 2)

Study measure	Range	Condition											
		Woman—Unspecified		Man—Intercourse Only		Man—Clitoral Stimulation		Man—Reliable Route					
		*Mean*	*SD*	*Mean*	*SD*	*Mean*	*SD*	*Mean*	*SD*	*F*	*dfs*	*p*	*η*_*p*_^2^
Orgasm value	1–7	4.35^a^	1.27	4.43^a^	1.36	4.83^a^	1.30	4.68^a^	1.21	2.67	3, 358	**.048**	0.02
Orgasm expectations	1–7	5.35^a^	1.25	3.95^b^	1.75	5.23^a^	1.51	5.28^a^	1.31	19.44	3, 358	** < .001**	0.14
Orgasm goal pursuit	1–7	5.39^a,b^	1.03	5.11^a^	1.22	5.71^b^	0.98	5.65^b^	0.97	6.18	3, 358	** < .001**	0.05
Pursuit of partner’s orgasm	1–7	6.41	0.57	6.44	0.59	6.45	0.55	6.48	0.53	0.22	3, 358	.884	0.00
Perceived partner orgasm pursuit	1–7	6.03^a^	0.69	4.93^b^	1.32	5.85^a^	0.87	5.95^a^	0.82	26.48	3, 358	** < .001**	0.18
Anticipated clitoral stimulation	1–7	5.65^a^	0.70	4.38^b^	1.21	5.19^c^	0.95	5.25^c^	1.02	27.50	3, 358	** < .001**	0.19
Sex acts													
Vaginal intercourse with simultaneous clitoral stimulation*^+^	1–7	5.18^a,b^	1.25	4.74^a^	1.44	5.37^b^	1.45	5.48^b^	1.36	5.10	3, 358	**.002**	0.04
Vaginal intercourse without simultaneous clitoral stimulation^+^	1–7	3.92^a^	1.54	5.52^b^	1.52	5.20^b^	1.59	5.25^b^	1.82	18.61	3, 358	** < .001**	0.14
Anal Intercourse^+^	1–7	2.26	1.39	1.96	1.11	2.24	1.47	2.49	1.48	2.36	3, 358	.072	0.02
Give oral sex	1–7	5.81^a^	1.20	5.09^b^	1.55	5.50^a,b^	1.16	5.52^a,b^	1.21	5.00	3, 358	**.002**	0.04
Receive oral sex*^+^	1–7	5.74^a^	1.04	4.26^b^	1.46	5.00^c^	1.58	5.18^c^	1.36	19.26	3, 357	** < .001**	0.14
Manual stimulation of partner’s genitals	1–7	6.32^a^	0.67	5.78^b^	1.10	6.09^a,b^	0.83	6.10^a,b^	0.83	5.98	3, 358	** < .001**	0.05
Receive manual stimulation of clitoris from partner*^+^	1–7	6.19^a^	0.78	4.91^b^	1.48	5.94^a^	0.91	5.78^a^	1.06	24.20	3, 358	** < .001**	0.17
Receive manual stimulation of vagina from partner^+^	1–7	6.17^a^	0.95	4.99^b^	1.50	5.83^a^	0.89	5.75^a^	1.03	18.65	3, 358	** < .001**	0.14
Gentle Kissing	1–7	6.32^a^	0.79	5.61^b^	1.25	5.82^b^	1.15	6.00^a,b^	1.01	7.54	3, 358	** < .001**	0.06
Deep Kissing	1–7	6.27^a^	0.89	5.73^b^	1.42	6.12^a,b^	1.04	6.13^a,b^	0.83	4.38	3, 358	**.005**	0.04
Use of a vibrator or other sex toy on clitoris*^+^	1–7	5.46^a^	1.27	3.60^b^	1.76	4.44^c^	1.58	4.58^c^	1.81	21.19	3, 358	** < .001**	0.15
Use of a vibrator or other sex toy in vagina^+^	1–7	5.09^a^	1.42	3.05^b^	1.48	3.91^c^	1.47	3.73^c^	1.67	30.21	3, 358	** < .001**	0.20
Use of a vibrator or other sex toy on partner	1–7	5.37^a^	1.21	2.54^b^	1.41	3.21^c^	1.47	3.33^c^	1.70	67.53	3, 357	** < .001**	0.36
Sexual touching or oral stimulation to body parts other than genitals (e.g., nipples, neck, thighs, etc.)^+^	1–7	6.48^a^	0.69	5.65^b^	1.32	6.26^a,c^	0.84	6.07^c^	1.20	10.86	3, 358	** < .001**	0.08
Sexual touching or oral stimulation to partner’s body parts other than genitals (e.g., nipples, neck, thighs, etc.)	1–7	6.51^a^	0.71	5.60^b^	1.22	5.96^b,c^	1.11	6.12^a,c^	1.09	12.47	3, 358	** < .001**	0.10
Stimulate partner’s anus with hands or mouth	1–7	2.93^a^	1.61	2.13^b^	1.26	2.18^b^	1.41	2.28^b^	1.37	6.51	3, 358	** < .001**	0.05
Receive anal stimulation from partner’s hands or mouth^+^	1–7	3.22	1.83	2.77	1.64	2.61	1.78	3.00	1.73	2.13	3, 358	.096	0.02
Partner would masturbate in from of me with their hands	1–7	4.77^a^	1.30	3.62^b^	1.53	3.89^b^	1.59	3.70^b^	1.53	12.07	3, 358	** < .001**	0.09
Masturbate in front of partner with hands^+^	1–7	4.69^a^	1.54	3.67^b^	1.47	4.18^a,b^	1.58	4.13^a,b^	1.75	6.70	3, 358	** < .001**	0.05

^*^Indicates the four items included in the clitoral stimulation composite. ^+^Indicates the eleven items that were included in the Reliable Routes to Orgasm measure. Bolded *p*-values indicate *p* < .05. For pairwise comparisons with a significant *F*-test, shared superscripts (a, b, c) indicate means which do *not* significantly differ, *p* > .05

## Results

### Most Reliable Route to Orgasm

Once again, the four items included in the clitoral stimulation composite measure were the four sex acts that were most commonly listed as participants’ most reliable route to orgasm (Table 3): use of a vibrator or other sex toy on your clitoris (72.7% of participants), vaginal intercourse with simultaneous clitoral stimulation (64.9%), receive oral sex from partner (48.3%), and receive manual stimulation of your clitoris from partner (47.8%). Again, there was a significant drop in frequency for the next most popular response (manual stimulation of your vagina from partner; 30.1%), *χ*^2^ (1, 362) = 20.85, *p* < 0.001.

### Differences by Condition

#### Anticipated Clitoral Stimulation

In Study 2, anticipated clitoral stimulation served as a manipulation check. Participants differed in their expectations for clitoral stimulation based on condition (see Table 5 for ANOVA results by condition). Women in the Man—Intercourse Only condition had lower expectations for clitoral stimulation than women in all other conditions. Anticipated clitoral stimulation did not differ between the Man—Clitoral Stimulation and Man—Reliable route conditions. Thus, we successfully manipulated clitoral stimulation in the interpersonal sexual script with a male partner. However, women still expected more clitoral stimulation in the Woman—Unspecified condition than in any of the three conditions with a male partner.

#### Perceived Partner Orgasm Pursuit

**Fig. 4:**
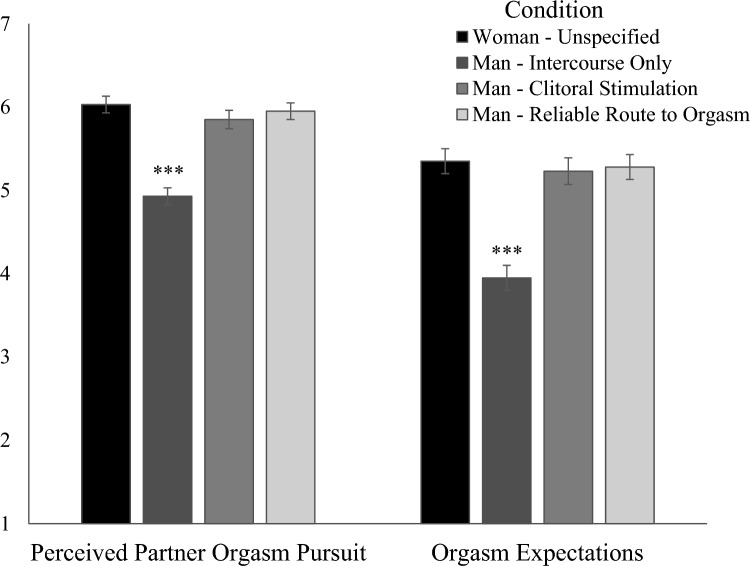
Differences in perceived partner orgasm pursuit and orgasm expectations by condition (Study 2). *Note* * *p* < .05; ** *p* < .01; *** *p* < .001. Error bars ± 1 Standard Error. The Man—Intercourse Only condition differed from all other conditions, which did not differ from each other

Women’s perceptions of their partner’s pursuit of their orgasm differed by condition (Fig. 4): Women in the Man—Intercourse Only condition reported significantly less perceived partner pursuit than those in the other three conditions, which did not differ from each other.

#### Orgasm Expectations

Women’s expectations for orgasm differed by condition (Fig. 4): Women’s expectations for orgasm were lower in the Man—Intercourse Only condition than in the other three conditions, which did not differ.

#### Additional Variables

While the initial *F*-test indicated that women’s orgasm value differed by condition, no pairwise comparisons reached statistical significance. Women did not differ in pursuit of their partner’s orgasm based on condition. However, women’s pursuit of their own orgasm differed by condition, with women in the Man—Intercourse Only condition reporting less pursuit of their own orgasm than women in the Man—Clitoral Stimulation and Man—Reliable Route conditions. The Woman—Unspecified condition did not differ from any other conditions for women’s own orgasm pursuit. See Table 5 for all ANOVA comparisons, including specific sex acts.

### Mediation Model

**Fig. 5:**
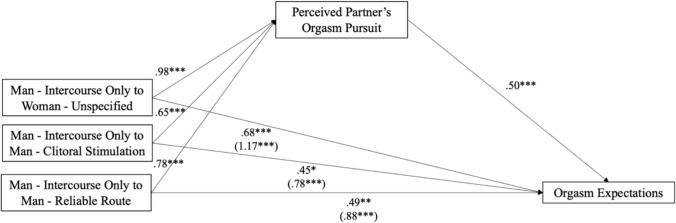
Mediation predicting orgasm expectations (Study 2). *Note* Indirect effect for Woman—Unspecified = .49 [.30, .69]. Indirect effect for Man—Clitoral Stimulation = .33 [.16, .52]. Indirect effect for Man—Reliable Route = .39 [.22, .58]. Analyses controlled for women’s own orgasm pursuit. Unstandardized values are shown

In our multi-categorical mediation model, we compared each condition separately to the Man—Intercourse Only condition, which was coded as the reference group. Using 5,000 bootstrapped samples, the mediation (i.e., indirect effect) was significant for each comparison—for the Woman—Unspecified condition, indirect effect = 0.49 [95% CI: 0.30, 0.69], *SE* = 0.10, for the Man—Clitoral Stimulation condition, indirect effect = 0.33 [95% CI: 0.16, 0.52], *SE* = 0.09, and for the Man—Reliable Route condition, indirect effect = 0.39 [95% CI: 0.22, 0.58], *SE* = 0.10. We found that condition significantly predicted perceived partner orgasm pursuit, such that being in the Man—Intercourse Only condition predicted less perceived partner orgasm pursuit compared to the other three conditions, *ps* < 0.001. Greater perceived partner pursuit predicted greater orgasm expectations, *p* < 0.001. See Fig. 5 for coefficients. Women’s own orgasm pursuit was a significant covariate, *ps* < 0.001.

### Discussion (Study 2)

In Study 2, we provided evidence that orgasm expectations with a male partner become commensurate to those with a female partner when manipulating the sexual script with a hypothetical male partner to include sufficient opportunity for orgasm via clitoral stimulation. As in Study 1, bisexual women expected orgasm when they expected more clitoral stimulation and greater partner’s pursuit of their orgasm. When anticipating a sexual encounter with men which included intercourse only, women expected their partner to pursue their orgasm less, and expected orgasm less as a result. When anticipating a sexual encounter with men which specified clitoral stimulation and opportunity for orgasm, however, bisexual women expected partner orgasm pursuit and orgasm equal to an unspecified sexual encounter with a woman. These findings underscore the influence and malleability of sexual scripts at the individual and interpersonal level (Wiederman, 2015). Particularly, while traditional sociocultural scripts for heterosexual couples tend to deprioritize clitoral stimulation, partners can incorporate these behavioral elements to increase women’s orgasm expectations, and, by extension, orgasm frequency in their own interpersonal relationships.

## General Discussion

The current project aimed to investigate the role and malleability of sexual scripts in informing partner gender differences in orgasm expectations. Using samples of bisexual women to experimentally isolate the effect of partner gender from sexual orientation on orgasm-relevant outcomes, we find that bisexual women report greater orgasm expectations when partnered with women versus men in hypothetical scenarios, due in part to greater expectations for clitoral stimulation and perceived partner orgasm pursuit when partnered with women versus men. However, when manipulating the interpersonal sexual script with a hypothetical man in Study 2, we find that women’s orgasm expectations when partnered with men can equal those when partnered with women if sufficient opportunity for orgasm via clitoral stimulation is specified in the sexual scenario with men. Thus, we conclude that the orgasm gap for women who have sex with men is due, in large part, to differences in the dominant sociocultural script when women have sex with men—specifically, insufficient clitoral stimulation and insufficient pursuit of women’s orgasm.

Women generally expect (1) more clitoral stimulation and (2) greater partner pursuit of their orgasm when having sex with women compared to men. Substantial variation still exists in women’s orgasm expectations even when clitoral stimulation is present, and additional work is needed to test whether interventions could effectively shift men’s behavior by helping couples’ revise their interpersonal sexual scripts. Wiederman (2005, 2015) suggests that couples can construct revised sexual scripts by explicitly writing out their typical sexual encounters and identifying elements they want to retain or replace to maximize both partners’ sexual needs. Our findings point to important elements that men partnered with women could incorporate into their revised sexual scripts to reduce this partner gender gap in expectations and resulting orgasm frequency: increased pursuit of women’s orgasm via clitoral stimulation.

### Sexual Scripts

These findings align with previous research that women experience the lowest orgasm frequency during sexual encounters that include intercourse alone (Frederick et al., 2018). Thus, a definition of “sex” which is limited to penile-vaginal intercourse (e.g., Randall & Byers, 2003) does not capture the sex acts that women generally need in order to experience orgasm. Because women had lower orgasm expectations when partnered with men than with women in Study 1, but equally once clitoral stimulation was specified in Study 2, these findings suggest that clitoral stimulation is not necessarily assumed to be part of the sociocultural sexual script during sex with men, or at least not *enough* clitoral stimulation is assumed in order to facilitate orgasm. Previous research has not yet established how often sexual encounters between men and women are restricted to vaginal intercourse only, though it does happen (e.g., Frederick et al., 2018). Specifying clitoral stimulation increased women’s expectations for orgasm with men to those when partnered with women, though variability in women’s orgasm expectation remains.

These results correspond with social scripting theory, which suggests that, while sociocultural scripts implicitly prescribe gender roles, normative behaviors, and expectations by default (as observed in Study 1), sexual partners can resist and reconstruct their interpersonal scripts to better align with their sexual needs and relational dynamics (Lamont, 2017; Masters et al., 2013; Wiederman, 2005)—for example normalizing clitoral stimulation and shared orgasm pursuit (as we did in Study 2). Considering that adaptations at the interpersonal level still occur within the constraints of broader sociocultural narratives that maintain gendered sexual expectations (e.g., Chadwick, 2024), the remaining variability in women’s orgasm expectations may represent the individual-level of scripting—one’s internal, intrapsychic experience negotiating the sociocultural and interpersonal (Simon & Gagnon, 1984, 1986; Wiederman, 2015).

In the typical heterosexual script, foreplay includes any sex acts beyond intercourse and is typically used to get the couple, particularly the woman, ready for intercourse (Mahar et al., 2020). Previous research has found that women having sex with women experience a longer duration of sex on average (30–45 min) than women having sex with men (15–30 min), and sex duration also predicts women’s orgasm frequency (Blair & Pukall, 2014; Frederick et al., 2018). This longer sex duration may be explained by the greater variety of sex acts typically experienced when women have sex with women compared to men (Blair & Pukall, 2014; Frederick et al., 2018). Previous research has found that heterosexual women report lower orgasm expectations and less intent to pursue orgasm when told that a sexual encounter will be quick than when told they have ample time (Wetzel et al., 2024). Thus, quick sexual encounters for heterosexual couples (i.e., “quickies”) likely contribute to the orgasm gap. Women in heterosexual partnerships may engage in “quickies” for a variety of reasons, such as to connect with their partner or manage disparate sexual desire (Blumenstock, 2022; Mark et al., 2014; Regan & Bersched, 1996; Willoughby et al., 2014). However, previous research has found that men and women both desire an average of around 18 min of foreplay (Miller & Byers, 2004), yet women having sex with men report an average sex duration of only 15–30 min in its entirety (Blair & Pukall, 2014). Thus, the average sexual experience between men and women may not include enough time for sex acts beyond intercourse to result in women’s orgasm.

### Interpersonal Orgasm Pursuit

These findings also emphasize the importance of interpersonal orgasm pursuit, or studying the pursuit of orgasm as a shared, interdependent process between partners (Wolfer & Carmichael, 2025). While women’s orgasm pursuit is an important predictor of women’s orgasm frequency, this pursuit is influenced by contextual (Dickman et al., 2024; Wetzel et al., 2024) and relational factors (Wolfer & Carmichael, 2025). Specifically, research finds that perceived partner pursuit is essential for one’s own orgasm pursuit, and that one’s effort toward orgasm is enhanced and more successful when met with perceived collaboration from a partner (Wolfer & Carmichael, 2025). Relatedly, women who read about a selfish partner report less intent to pursue orgasm than women who read about a partner who appears invested in her pleasure (Wetzel et al., 2024).

The current research adds to this literature by investigating perceived partner orgasm pursuit as a mechanism for predicting orgasm expectations, and finds that women expect orgasm more during sexual encounters that include sufficient clitoral stimulation, in part, because they perceive their partner to be invested in and pursuing their orgasm. Perceived partner orgasm pursuit explains unique variance in women’s orgasm expectations even when controlling for women’s own orgasm pursuit, demonstrating how orgasm outcomes are a relational rather than individual process. Our research finds that male partners’ prioritization of clitoral stimulation in the sexual script can increase perceptions of orgasm pursuit, which subsequently increases women’s orgasm expectations.

### Implications

Orgasm expectations provide a useful proxy measure for orgasm in studies using a hypothetical design, as orgasm expectations and orgasm frequency are highly correlated (e.g., Dickman et al., 2024). Orgasm expectations also directly predict women’s orgasm pursuit (Dickman et al., 2024; Wetzel et al., 2024). By examining predictors of orgasm expectations, this research sheds light on mechanisms that can be used to address the orgasm gap. By highlighting the instrumental role of partner gender, this project also has implications for understanding how partner gender may influence other gender differences (e.g., interest in casual sex, sexual desire), providing applications for sexuality and gender research more broadly.

Our findings inform steps we can take at both the sociocultural and interpersonal level to eliminate orgasm and pleasure disparities. At the sociocultural level, it would be useful to raise awareness of sexual scripts and how they contribute to gendered orgasm disparities. Sex education, sex therapy, and social marketing campaigns can, for example, seek to normalize new sociocultural scripts that prioritize clitoral stimulation and pursuit of women’s orgasm. Such sociocultural changes require long-term structural efforts and are less malleable than interpersonal scripts, which are iteratively modified and co-constructed by partners (Simon & Gagnon, 1984; Simon & Gagnon, 1986; Wiederman, 2005; Wiederman, 2015).

To facilitate change at the interpersonal level, men having sex with women may choose to focus less on intercourse as the main sex act and more on pursuing their partner’s orgasm via sufficient clitoral stimulation, beyond a few minutes of “foreplay.” Since individual variability remains among women’s orgasm expectations in general and from specific sex acts, couples should incorporate the sex acts that are most pleasurable for the female partner. Our research shows that the most reliable routes to orgasm can differ between women, but consistently involve clitoral stimulation. By incorporating the sex acts that a given woman needs in order to orgasm, men can demonstrate to their female partners that they are actively invested in and pursuing her orgasm. Men should collaborate with their female partners on the pursuit of her orgasm (Wolfer & Carmichael, 2025), and sexual communication can help reveal her specific preferences so, together, they can hone in on the right combination of sex acts to maximize pleasure (Jones et al., 2018; Mallory et al., 2019). The current work also demonstrates the need for more research and attention on interdependent orgasm pursuit as an important mechanism in orgasm equality.

In sum, these findings suggest that couples should work together to (1) incorporate more clitoral stimulation, (2) enthusiastically pursue women’s orgasm, and (3) personalize orgasm pursuit to each individual, if they wish to increase women’s orgasm frequency.

### Limitations and Future Directions

There are some important limitations to the current research. First, the sexual scenarios utilized in these studies are completely hypothetical, and thus do not capture the complexity of sexual relationships. However, hypothetical scenarios allow researchers to utilize an experimental design with empirical control that would otherwise not be possible. These results should be replicated, however, in a design that compares women’s experiences with real partners across genders. Future research should also fully cross-conditions by partner gender, to demonstrate how women would react to female partners in the same sexual script conditions that were presented for male partners.

Additionally, the relationship context in the sexual scenarios was intentionally vague and was designed to mimic the context of a “familiar” partner used in existing literature (e.g., Garcia et al., 2014; Wetzel & Sanchez, 2022). However, this vague relationship context could have been interpreted differently by participants (e.g., a new dating relationship versus a six-month committed relationship), which may have influenced participants’ responses.

While the intercourse only condition in Study 2 was meant to mimic a script with a primary focus on penile-vaginal intercourse, some participants may have interpreted this script as the complete absence of any foreplay, including kissing, making this condition potentially unrealistic or, at minimum, difficult to interpret. Based on our data from Study 2, however, participants still expected other sex acts to occur in this condition, but they expected them less than participants in other conditions. Thus, future research should investigate what “quickies” or intercourse-focused sexual encounters actually look like for mixed-sex couples, as well as how often they occur (Frederick et al., 2018). Also, in Study 2, the reliable route condition likely indicated more than just the addition of clitoral stimulation, but a male partner who is receptive and dedicated to the participant’s pleasure. Future research should better isolate specific variables to determine what qualities of a sexual partner, encounter, or script best drive differences in orgasm expectations.

Relatedly, the present research successfully changed expectations for a given encounter and altered sexual scripts by explicitly changing aspects of hypothetical sexual scenarios. However, it remains unknown whether we did (and can) change someone’s default sexual script, which is often psychologically internalized and socially conditioned. Future research ought to test whether being partnered with a man who is regularly attentive can change a woman's default script, and whether teaching or encouraging equitable sexual scripts can effectively change men’s (and women’s) default scripts.

Additionally, variance remained in anticipated clitoral stimulation and orgasm expectations when women have sex with other women versus men that was not explained by the variables included in our study. Thus, other elements of sexual encounters with women that predict orgasm were not captured in the current project. For example, women may find it easier to communicate about their sexual needs with other women compared to men (e.g., Bell & Weinberg, 1978). Additionally, sexual minorities are less likely than heterosexual people to be strictly tied to any specific script (Lamont, 2017), which may reflect sexual adaptability (Fournier et al., 2023). In our Study 2 results, women still expected some sex acts to be more likely with women than with men across conditions (e.g., receiving oral sex, vibrator use, sexual touching of other body parts). These findings supplement existing research that the sexual script for women having sex with women tends to incorporate more sexual variety than the script with men (Frederick et al., 2018). Thus, even when clitoral stimulation is specified, there are still differences in default sexual scripts when women have sex with women versus men. Future research should investigate these and other potential mechanisms to continue to explain the orgasm gap for women across partner gender and sexual orientation groups.

Finally, these results do not necessarily generalize to all groups of women. The samples included in this project were majority-White, bisexual and pansexual, and only included cisgender women. The experiences of bisexual and/or pansexual women may be distinct from heterosexual and lesbian women’s experiences in ways that were not assessed here (e.g., Fournier et al., 2023; Thöni et al., 2024). However, the current research establishes important mechanisms for predicting women’s orgasm, which previous research suggests to be generalizable across racial/ethnic and sexual orientation groups (Dickman et al., 2024; Thorpe et al., 2024; Townes et al., 2022; Wetzel et al., 2024, 2025).

### Conclusion

In sum, women expect orgasm to be more likely when they expect clitoral stimulation and partner pursuit of their orgasm. While these two mechanisms are anticipated more with a female partner than a male partner, this partner gender effect can be eliminated when we shift the sexual script within a given scenario to raise expectations for clitoral stimulation and pursuit of women's orgasm with a hypothetical male partner. Thus, these results suggest that partner gender and sexual orientation differences in orgasm frequency are, in part, due to differences in the sociocultural sexual script when women have sex with other women versus men. We identify clitoral stimulation and interdependent pursuit of women’s orgasm as two key mechanisms for addressing the orgasm gap. At the interpersonal level, sexual scripts are flexible and can be changed; thus, men partnered with women could increase women’s orgasm expectations and subsequent frequency by actively pursuing their partner’s orgasm via clitoral stimulation, in collaboration with their partner.

## Supplementary Information

Below is the link to the electronic supplementary material.

Supplementary file1 (DOCX 18 KB)

## Data Availability

Data and materials are available at https://osf.io/qhd5n/.

## Code Availability

Syntax files used with SPSS software are available at https://osf.io/qhd5n/.
