# Supplementary material for: An Experimental Investigation of Sexual Scripts by Partner Gender: Anticipated Clitoral Stimulation and Partner Orgasm Pursuit Shape Women’s Orgasm Expectations
Source: Arch Sex Behav. 2025 Jun 17;54(6):2167–84. doi: 10.1007/s10508-025-03169-4 (PMC12283801; doi:10.1007/s10508-025-03169-4)
Supplement: Supplementary file 1 — Supplementary file1 (DOCX 18 KB) [file 10508_2025_3169_MOESM1_ESM.docx]

**An experimental investigation of sexual scripts by partner gender: Anticipated clitoral stimulation and partner orgasm pursuit shape women’s orgasm expectations**

Supplement Document

**Differences by Data Source**

**Study 1**

We tested for differences in age, real-life orgasm experiences, and orgasm expectations in response to the vignette (i.e., our main outcome variable) by data source: between participants who were recruited from ResearchMatch versus Prolific. In Study 1, there were differences in age between the samples, with the ResearchMatch sample being older (*M* = 34.7 years) than the Prolific sample (*M* = 31.1 years), *p* < .001. However, bisexual women from both samples reported equal orgasm frequencies in their real life, *p* = .893, and orgasm expectations in response to the vignette, *p* = .722.

**Study 2**

For Study, 2, there was no difference in age between the ResearchMatch (*M* = 33.8 years) and Prolific samples (*M* = 32.8 years), *p* = .388. There was no difference in participants’ real life orgasm frequencies, *p* = .933. However, ResearchMatch participants expected orgasm (*M* = 4.8) less than Prolific participants did (*M* = 5.3) in response to the vignette, *p* = .002. This outcome may be informed by Prolific participants’ greater expectation for clitoral stimulation in response to the vignettes (*M* = 5.3) compared to ResearchMatch participants (M = 5.0), *p* = .028. Perceived partner orgasm pursuit did not differ by data source, *p* = .172. Future research ought to investigate the demographic (or other) variable(s) that contribute to the observed difference in vignette expectations by data source.

**Pilot Version of Study 2**

**Sample & Procedure**

After eligibility and exclusions, we collected a final sample of 264 participants from the Prolific recruitment platform. Participants had to be cisgender, bisexual or pansexual women, at least 18 years old, residing in the United States. After completing screening, participants were randomly assigned to one of four conditions, were asked to read a hypothetical sexual scenario, and completed the measures below in the order presented. Finally, participants completed demographic questions and received debriefing.

**Measures**

Pilot vignettes with a male partner for Study 2 included a “traditional script” condition in which vaginal intercourse was the main sex act, a “clitoral stimulation” condition in which clitoral stimulation was specified, and an “opportunity for orgasm” condition in which opportunity for orgasm was specified, without referencing clitoral stimulation or any specific sex act.

Participants were given the same instructions as in Study 1, and were randomly assigned to read one of four vignettes. The “Woman – Comparison” condition was exactly the same as the female partner condition used in Study 1. The “Man – Traditional Script” condition included an additional sentence specifying details about the sexual script with the hypothetical partner, reflecting the prioritization of vaginal intercourse in the typical sexual script for heterosexual partnered sex. The additional sentence is presented here in bold text:

“You are out to dinner with a man who you feel comfortable with and find very attractive. You’ve been seeing this man for a while and have an established sexual relationship with him. When you get home from dinner, things start heating up and you make your way to the bedroom together. **In the past, you and this partner have typically engaged in vaginal intercourse as the main sexual activity during your sexual encounters.** You are in the mood and are looking forward to beginning sexual activity with him.”

The “Man – Clitoral Stimulation” condition included the following sentence instead: “In the past, you and this partner have typically engaged in a range of sexual activities in your sexual encounters, always including clitoral stimulation.” The “Man – Opportunity for Orgasm” condition included the following sentence instead: “In the past, you and this partner have typically engaged in a range of sexual activities in your encounters to ensure you both have the opportunity to orgasm.”

Participants were asked to complete the same additional measures as in the final version of Study 2, keeping in mind the sexual encounter described, including three orgasm goal pursuit measures, orgasm expectations, orgasm value, and sex acts. The clitoral stimulation composite was made using the same four sex acts as in Studies 1 and 2. At the end of the study, we administered a manipulation check via two questions to ensure that participants could correctly identify the vignette they were assigned. First, we asked participants to identify the gender of their hypothetical partner. Then, for participants assigned to one of the three male partner conditions, we presented a list of three condition-specific sentences and asked them to select the one that appeared in their scenario. Only participants who responded to these questions correctly were included in the final sample.

**Results**

***Clitoral Stimulation***

We attempted to manipulate opportunity for orgasm in the sexual script, via clitoral stimulation, with male partners specifically, as compared to a female partner comparison. There was a difference in expected clitoral stimulation by condition, *F*(3, 260) = 7.87, *p* < .001, η_p_^2^ = .08. Women reported greater expectations for clitoral stimulation in the woman comparison condition (*M* = 5.63, *SD* = 0.71) than the man traditional script (*M* = 5.02, *SD* = 0.99) and man clitoral stimulation (*M* = 4.93, *SD* = 1.00) conditions, *ps* < .001. Women in the man opportunity for orgasm condition (*M* = 5.24, *SD* = 0.96) did not differ from women in the woman comparison condition, *p* = .061. However, none of the male partner conditions differed in expected clitoral stimulation, *ps* > .403. Thus, we did not successfully manipulate clitoral stimulation in the sexual script between our three conditions with hypothetical male partners.

We ran analyses again on a composite of three of the four clitoral stimulation items, excluding vibrator use, in case the vibrator use item was skewing the results with a male partner. There was a difference in expected clitoral stimulation by condition, *F*(3, 260) = 3.69, *p* = .012, η_p_^2^ = .04. Women expected more clitoral stimulation with a woman (*M* = 5.76, *SD* = 0.79) than with a man in the clitoral stimulation condition (*M* = 5.25, *SD* = 1.04), *p* = .016. None of the male partner conditions differed in expected clitoral stimulation, *ps* > .397 – not the traditional script condition (*M* = 5.37, *SD* = 0.96) nor the opportunity for orgasm condition (*M* = 5.57, *SD* = 1.01). Again, we did not successfully manipulate clitoral stimulation in the sexual script between male partner conditions.

**Interpretation & Discussion**

We did not successfully manipulate what we attempted to (i.e., clitoral stimulation in the sexual script with men) in the pilot version of this project. In the traditional sexual script with a male partner, clitoral stimulation is typically included during brief foreplay (Mahar et al., 2020). Thus, these sexual acts (e.g., manual stimulation) may be likely to occur in a traditional script which prioritizes intercourse. However, there is not *enough* clitoral stimulation included in this prototypical script to facilitate women’s orgasm. Thus, we suspect that there was no substantial difference between the “traditional script” where intercourse was prioritized and the script where clitoral stimulation was mentioned, both theoretically and in our data. For women partnered with men, “vaginal intercourse as the main sexual activity” and “including clitoral stimulation” appear to evoke a similar sexual script, at least in terms of the clitoral sex acts included in that script. The “opportunity for orgasm” condition came closer to successfully manipulating women’s expectations for the encounter, as expected clitoral stimulation in this condition did not significantly differ from the woman comparison condition. However, expected clitoral stimulation did not differ between *any* of the three conditions with a male partner, indicating an unsuccessful manipulation.

In the revised version of the vignettes (Study 2), we clearly differentiated the conditions according to the following three categories: intercourse alone, consistent clitoral stimulation, and opportunity to orgasm via one’s most reliable route. Thus, in the final version, we attempted to establish a difference between an intercourse-based script and a script in which general clitoral stimulation and/or one’s most reliable route to orgasm is more clearly prioritized.
